# Systematic review and individual participant data meta-analysis of randomized controlled trials assessing mindfulness-based programs for mental health promotion

**DOI:** 10.1038/s44220-023-00081-5

**Published:** 2023-07-10

**Authors:** Julieta Galante, Claire Friedrich, Collaboration of Mindfulness Trials (CoMinT), Napaporn Aeamla-Or, Marieke Arts-de Jong, Bruce Barrett, Susan M. Bögels, Jan K. Buitelaar, Mary M. Checovich, Michael S. Christopher, Richard J. Davidson, Antonia Errazuriz, Simon B. Goldberg, Corina U. Greven, Matthew J. Hirshberg, Shu-Ling Huang, Matthew Hunsinger, Yoon-Suk Hwang, Peter B. Jones, Oleg N. Medvedev, Melissa A. Rosenkranz, Melanie P. J. Schellekens, Nienke M. Siebelink, Nirbhay N. Singh, Anne E. M. Speckens, Feng-Cheng Tang, Lianne Tomfohr-Madsen, Tim Dalgleish, Peter B. Jones, Ian R. White

**Affiliations:** 1 Department of Psychiatry, University of Cambridge, Cambridge, United Kingdom; 2 Contemplative Studies Centre, Melbourne School of Psychological Sciences, Faculty of Medicine, Dentistry, and Health Sciences, University of Melbourne, Melbourne, Australia; 3 MRC Cognition and Brain Sciences Unit, University of Cambridge, Cambridge, United Kingdom; 4 Cambridgeshire and Peterborough NHS Foundation Trust, Cambridge, UK; 5 NIHR Applied Research Collaboration East of England, Cambridge, UK; 6 MRC Clinical Trials Unit at UCL, University College London, London, United Kingdom; 7 School of Nursing, University of Phayao, Phayao, Thailand; 8 Radboudumc Centre for Mindfulness, Department of Psychiatry, Radboud University Medical Centre, Nijmegen, The Netherlands; 9 Department of Family Medicine and Community Health, University of Wisconsin-Madison, Madison, WI USA; 10 Department of Developmental Psychology, University of Amsterdam, Amsterdam, The Netherlands; 11 Research Institute of Child Development and Education, University of Amsterdam, Amsterdam, The Netherlands; 12 Donders Institute for Brain, Cognition and Behaviour, Department of Cognitive Neuroscience, Radboud University Medical Centre, Nijmegen, The Netherlands; 13 Karakter Child and Adolescent Psychiatry University Center, Nijmegen, The Netherlands; 14 School of Graduate Psychology, Pacific University, Hillsboro, OR USA; 15 Center for Healthy Minds, University of Wisconsin-Madison, Madison, WI USA; 16 Department of Psychology, University of Wisconsin-Madison, Madison, WI USA; 17 Waisman Laboratory for Brain Imaging and Behavior, University of Wisconsin-Madison, Madison, WI USA; 18 Department of Psychiatry, University of Wisconsin-Madison, Madison, WI USA; 19 Department of Psychiatry, School of Medicine, Pontificia Universidad Católica de Chile, Santiago, Chile; 20 Millennium Institute for Research on Depression and Personality (MIDAP), Santiago, Chile; 21 Department of Counselling Psychology, University of Wisconsin-Madison, Madison, WI USA; 22 Department of Psychology, Chung Shan Medical University, Taichung, Taiwan; 23 Room of Clinical Psychology, Chung Shan Medical University Hospital, Taichung, Taiwan; 24 Institute for Learning Sciences and Teacher Education, Australian Catholic University, Brisbane, Queensland Australia; 25 School of Psychology, University of Waikato, Hamilton, New Zealand; 26 Department of Psychiatry, University of Wisconsin-Madison School of Medicine and Public Health, Madison, WI USA; 27 Academy Het Dorp, Arnhem, The Netherlands; 28 Department of Psychiatry and Health Behavior, Medical College of Georgia, Augusta University, Augusta, GA USA; 29 Department of Post-Baccalaureate Medicine, College of Medicine, National Chung Hsing University, Taichung, Taiwan; 30 Department of Occupational Medicine, Changhua Christian Hospital, Changhua, Taiwan; 31 Department of Leisure Services Management, Chaoyang University of Technology, Taichung, Taiwan; 32 Faculty of Education, University of British Columbia, Vancouver, British Columbia Canada; 33 Present Address: Scientific Research Department, Helen Dowling Institute, Centre for Psycho-Oncology, Scientific Research Department, Bilthoven, the Netherlands

**Keywords:** Lifestyle modification, Psychology

## Abstract

Mindfulness-based programs (MBPs) are widely used to prevent mental ill health. Evidence suggests beneficial average effects but wide variability. We aimed to confirm the effect of MBPs and to understand whether and how baseline distress, gender, age, education, and dispositional mindfulness modify the effect of MBPs on distress among adults in non-clinical settings. We conducted a systematic review and individual participant data (IPD) meta-analysis (PROSPERO CRD42020200117). Databases were searched in December 2020 for randomized controlled trials satisfying a quality threshold and comparing in-person, expert-defined MBPs with passive-control groups. Two researchers independently selected, extracted and appraised trials using the revised Cochrane Risk-of-Bias tool. IPD of eligible trials were sought from authors. The primary outcome was psychological distress (unpleasant mental or emotional experiences including anxiety and depression) at 1 to 6 months after program completion. Data were checked and imputed if missing. Pairwise, random-effects, two-stage IPD meta-analyses were conducted. Effect modification analyses followed a within-studies approach. Stakeholders were involved throughout this study. Fifteen trials were eligible; 13 trialists shared IPD (2,371 participants representing 8 countries. In comparison with passive-control groups, MBPs reduced average distress between 1 and 6 months post-intervention with a small to moderate effect size (standardized mean difference, −0.32; 95% confidence interval, −0.41 to −0.24; *P* < 0.001; no heterogeneity). Results were robust to sensitivity analyses and similar for the other timepoint ranges. Confidence in the primary outcome result is high. We found no clear indication that this effect is modified by the pre-specified candidates. Group-based teacher-led MBPs generally reduce psychological distress among volunteering community adults. More research is needed to identify sources of variability in outcomes at an individual level.

## Abstract (web summary)

Galante et al. performed a systematic review and meta-analysis using individual participant data from randomized controlled trials evaluating mindfulness-based programs for adult mental health promotion in non-clinical settings.

## Main

Depression and other common mental health disorders are among the leading global causes of morbidity, generating a substantial societal burden^1^. In 2015, 4.4% of the global population was estimated to be suffering from depression and 3.6% was estimated to have anxiety disorders^2^. The prevalence of anxiety and depression had increased by 14.9% and 18.4%, respectively, from 2005 to 2015 (ref. ^3^), despite the increase in the provision of treatment for common mental health disorders^4^. The COVID-19 pandemic has introduced new challenges to global mental health, particularly amongst at-risk individuals such as healthcare workers^5^, and there are concerns that these will persist beyond the pandemic period, without the right prevention and management approaches^6^. In general, there is widespread agreement that not enough emphasis is placed on prevention compared with the treatment of disorders^7^, and shifting to the implementation of preventive interventions must be done with care as they should be evidence based and delivered to a high standard of quality^4^.

The past decade has seen an expansion of mental health prevention and promotion programs in workplaces, educational establishments and other community settings^8^. Typically, they target psychological distress, a concept encompassing a range of disturbing or unpleasant mental or emotional experiences that usually include depression and anxiety^9^. Psychological distress is often an internal response to external stressors when coping mechanisms are overwhelmed. It is also frequently referred to as mental distress, emotional distress or simply stress. If unaddressed, psychological distress can result in mental and physical health disorders^10^.

MBPs are among the most popular preventive interventions^11^. It is estimated that 15% of British adults and 20% of Australians have practiced mindfulness meditation at some point in their lives; 5% in the United States have done so in 2017 (refs. ^12,13^). Mindfulness training is offered in over 600 companies globally^14^ and 79% of US medical schools^15^. National health guidelines in England encourage workplaces to help employees access mindfulness, yoga or meditation for their mental well-being^16^. During the COVID-19 pandemic, international mental health guidelines have been advocating for mindfulness training exercises^6,17^.

In these contexts, mindfulness is typically defined as “the awareness that emerges through paying attention on purpose, in the present moment, and nonjudgmentally to the unfolding of experience moment by moment”^18^. Core MBP elements are mindfulness meditation training, doing things mindfully such as eating or brushing one’s teeth, and collective and individual inquiry with a qualified teacher, using participatory learning processes^19^. MBPs emphasize scientific approaches to health and aim to be suitable for delivery in public institutions in various settings and across cultures.

We recently published a systematic review and aggregate-data (AD) meta-analysis of randomized controlled trials (RCTs) evaluating MBPs in non-clinical settings^20^. We found that MBPs reduce adults’ average psychological distress compared with no intervention. In our AD meta-analysis, we have also assessed whether effects vary as a function of study-level differences, such as MBP type and intensity. However, preliminary evidence strongly suggests that the effectiveness of MBPs varies as a function of individual, participant-level differences. Thus, there is a need for studies with sample sizes large enough to study such differences properly^21–27^. With the current surge of MBP use, it is crucial to move beyond simply studying group-level intervention effects and assess individual variability in MBP training responsiveness^28^. This could allow MBPs to be targeted at subgroups that will benefit most, maximizing effectiveness and cost-effectiveness and minimizing harm^29^.

## Individual-level factors

One individual-level factor with promising preliminary evidence for modifying MBP effects is pre-intervention psychological distress. Those who start with worse mental health may be the most likely to benefit from MBPs possibly because they tend to have more room for improvement and more motivation. There is evidence that MBPs targeted at stressed, anxious or symptomatic groups have larger effects^20,30–33^. In clinical settings, our IPD meta-analysis of mindfulness-based cognitive therapy (MBCT) to prevent recurrent depression relapse has found that those with worse baseline mental health would benefit more^34^. Another analysis combining three clinical trials has found a similar interaction effect^35^. However, some studies have found no evidence of such an interaction^36^.

Gender-specific effects may also be present in psychosocial interventions to promote health^37^. Some evidence from RCTs and AD meta-analyses suggests that MBPs’ effects on men are smaller than those on women, and multiple explanations for this have been proposed^26,38–40^. However, other studies, including our MBCT IPD meta-analysis, have found no significant influence of gender^30,34^.

A comparison of meta-analyses of MBPs for children and students with those of MBPs for adults suggests that effects may be larger among younger people^20,41–43^. Although some studies support this suggestion^44^, age was not a significant effect modifier in the clinical MBCT IPD meta-analysis nor in other studies^34,45^. Older people may be more engaged in training and therefore more likely to benefit.

The effects of some psychological interventions are known to be moderated by education levels^37,46^. There are concerns that those with lower education levels may not benefit equally from MBPs because of their language and cultural references^47^. A meta-analysis has recently shown that highly educated participants benefited more from a workplace MBP than others^38^. However, education levels did not significantly modify the effect of MBCT in the IPD meta-analysis with clinical populations^34^.

Another candidate effect modifier is dispositional mindfulness, a construct reflecting an individual’s focus and quality of attention^48^. Dispositional mindfulness, although very frequently measured, is an inconsistent concept, and it is unclear to what extent changes in dispositional mindfulness are specific to MBPs^49–52^. A higher level of dispositional mindfulness may be needed to engage with MBPs, but this may also limit the amount that is gained^28^. Some studies found that those with greater baseline dispositional mindfulness experienced greater mental health and well-being improvements after having participated in MBPs^27,45,53^.

## Rationale and aims of the study

So far, RCTs assessing MBPs in non-clinical settings have lacked the sample sizes needed to have adequate statistical power to assess individual differences in the effects of MBPs. Combining trials in meta-analyses has solved the sample size problem, but standard meta-regressions and subgroup meta-analyses are unable to avoid aggregation bias. This bias, sometimes referred to as ecological bias or fallacy, occurs when associations between average participant-level characteristics such as gender and the pooled intervention effect do not necessarily reflect the true associations between the participant-level characteristics and the intervention effect^54^. For example, a standard meta-regression may show that trials with a smaller mean age have a larger effect, but if these trials also happen to deliver longer MBPs, the larger effect is attributed to the delivery of longer MBPs rather than to having younger participants. Aggregation bias is avoided when interactions are examined at the participant level (that is, based on within-trial information).

As it allows for effect modification testing at the participant level, IPD meta-analysis is regarded as the ideal approach for estimating the modification effects of individual differences^22,23,54–57^. This approach is a specific type of systematic review that involves the collection, checking and re-analysis of the original data for each participant in each study^58^. This supports better-quality data and analysis, allowing for in-depth explorations and robust meta-analytic results, which may differ from those based on AD^59^.

In summary, IPD allow researchers to explore how intervention effects vary as a function of individual differences without aggregation bias, and the combination of data from multiple studies increases the power to detect such variations. IPD meta-analysis is also ideal for estimating intervention effects because the data can be checked and re-analyzed consistently across all the included samples, RCT data unused in previous analyses can be included, and missing data can be accounted for at the individual level^60,61^.

We therefore conducted the first, to our knowledge, systematic review and IPD meta-analysis of MBPs for adults in non-clinical settings. We aimed to estimate the effect of MBPs on psychological distress and to compare this with the results of our AD meta-analysis. In addition, crucially, we wanted to answer the following research question: Do our prespecified participant-level characteristics modify the effect of MBPs on psychological distress and, if so, how? Based on previous evidence, existing theories, the likelihood of availability of RCT data, and international comparability, our prespecified candidate effect modifiers were baseline psychological distress, gender, age, education and dispositional mindfulness.

## Results

### Study selection and characteristics

**Fig. 1:**
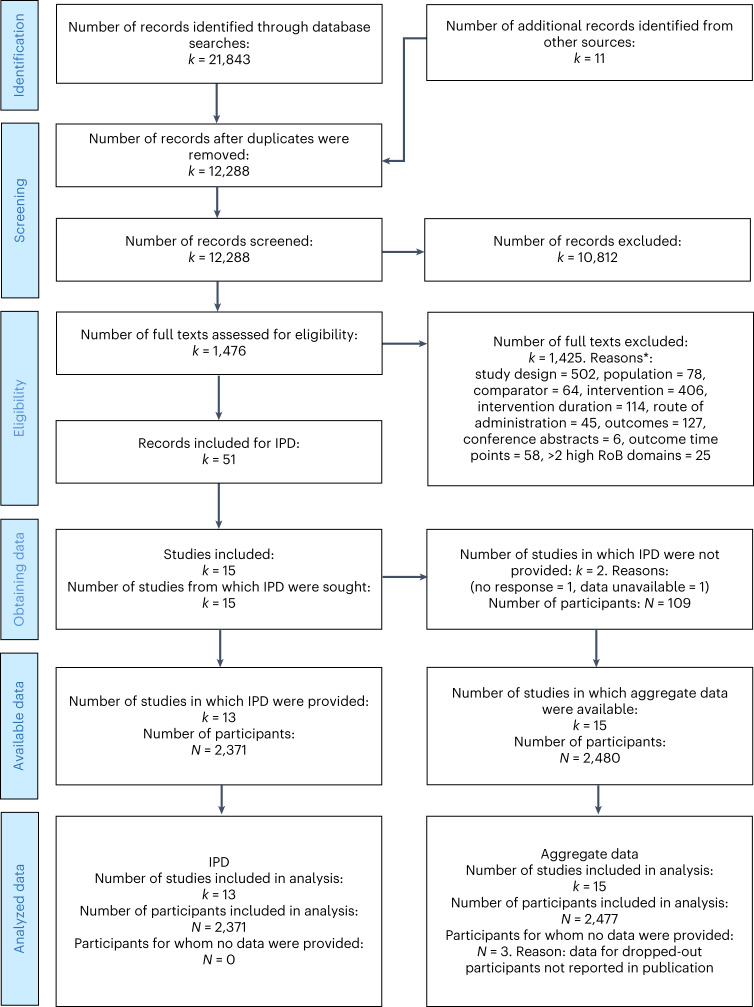
PRISMA IPD flow diagram. Reproduced with permission from the PRISMA IPD group, which encourages sharing and reuse for non-commercial purposes. *This section was built using a heuristic for quick study selection, whereby the easiest-to-assess criterion, study design, was assessed first and only the studies satisfying this criterion would be assessed for the subsequent criterion, and so on.

Figure 1 presents the IPD-specific Preferred Reporting Items for Systematic reviews and Meta-Analyses (PRISMA) study selection flow diagram. Combining our database searches from our previous review^20^ with the updated search, we obtained 21,843 records. We identified 11 additional records from other sources. Selection led to 51 records belonging to 15 trials deemed eligible sources of IPD.

Data were considered unavailable from 2 of the 15 eligible trials from which IPD were sought owing to the non-response to multiple contact attempts^62^ and the authors’ confirmation that data were inaccessible^63^. Therefore, only the IPD from 13 studies were included, amounting to 2,371 participants. This means we were able to obtain the IPD of 96% of the eligible participants and 87% of the eligible trials. The obtained IPD included two doctoral theses that did not have published results yet that were of interest to this review^64,65^. Five of the trials required a data transfer agreement to be in place before the authors could share the data. Throughout the process of obtaining and analyzing the data and completing the risk-of-bias assessments, the authors were contacted for any clarifications; nine authors were contacted about the data files and formats, and eight were contacted with queries regarding risk of bias.

The main study characteristics are summarized in Table 1. Of the 13 trials included, 4 were cluster RCTs. Publication dates ranged from 2012 to 2022; for two trials^66,67^, the main papers were published after our search, but their protocols had been identified in the search. Studies were conducted across eight countries: Australia, Canada, Chile, Taiwan, Thailand, the Netherlands, the United Kingdom and the United States.

**Table 1:** Characteristics of the included studies

Author, year, trial registration (if available), country	*N*	Participant type	Age, mean (s.d.)	Female (%)	Intervention	Control(s)	Distress measure	Follow-up timepoints	Effect modifiers
Aeamla-Or 2015^64^, ISRCTN62401721, Thailand	127	Nursing students	19.17 (0.87)	91	Mindfulness-based stress resilience	Treatment as usual from the University Mental Health Counselling Centre	PSS-10	Post-int, 2m, 6m	Mindfulness (MAAS), gender, age, education
Barrett 2012^104–110^, NCT01057771, United States	154	Older adults	59.27 (6.59)	82	Mindfulness meditation	(1) Waitlist, (2) exercise program	PSS-10	Post-int, 3m	Mindfulness (MAAS), gender, age, education
Barrett 2018^111–116^, NCT01654289, United States	413	Adults aged 30 to 69 years	49.65 (11.57)	76	MBSR	(1) No intervention, (2) progressive moderate-intensity exercise	PSS-10	Post-int, 2m, 3m, 6m	Mindfulness (MAAS), gender, age, education
Christopher 2018^117–119^, NCT02521454, United States	61	Law enforcement officers	43.97 (6.05)	11	Mindfulness-based resilience training	Waitlist	OPS	Post-int, 3m	Mindfulness (FFMQ-15), gender, age, education
Errazuriz 2020^120^, ISRCTN12039804, Chile	105	Healthcare professionals	40.16 (11.71)	97	MBSR	(1) Waitlist, (2) stress management course	PSS-14	Post-int, 4m	Mindfulness (FFMQ-39), gender, age, education
Galante 2018^39,121–123^, ACTRN12615001160527, United Kingdom	670	University students	23.5 (5.46)	64	Mindfulness skills for students plus mental health support as usual	Mental health support as usual	CORE-OM	Post-int, 1–4m, 10m	Gender, age, education
Huang 2015^124^, NCT02241070, Taiwan	144	Employees	42.54 (8.63)	41	Mindfulness-based intervention	Waitlist	PSS-10	Post-int, 1m, 2m	Gender, age, education
Hwang 2019^125^, Australia	185	School teachers	43.08 (11.59)	86	Reconnected	Waitlist	PSS-10	Post-int, 1.5m	Mindfulness (FFMQ-SF18), gender, age
Kral 2019^65,126–128^, NCT02157766, United States	139	Adults aged 25 to 65 years	44.11 (12.68)	59	MBSR	(1) Waitlist, (2) health enhancement program	SCL-90-R	Post-int, 6m	Mindfulness (FFMQ-39), gender, age, education
MacKinnon 2021^66,129^, NCT02214732, Canada	60	Pregnant women	31.75 (4.94)	100	MBCT for perinatal depression + treatment as usual	Treatment as usual	PSS-10	Post-int, 3m postpartum	Mindfulness (FFMQ-SF24), gender, age, education
Schellekens 2017^30,130,131^, NCT01494883, the Netherlands	44	Partners of patients with lung cancer	58.58 (9.63)	57	MBSR + care as usual	Care as usual	HADS	Post-int, 3m	Mindfulness (FFMQ-39), gender, age, education
Siebelink 2021^67,132^, NCT03220308, the Netherlands	102	Parents of children with ADHD	43.38 (5.47)	69	MYmind + care as usual	Care as usual	DASS-21	Post-int, 2m, 6m	Mindfulness (IM-P), gender, age, education
van Dijk 2017^71,133^, the Netherlands	167	Medical undergraduates	23.13 (2.55)	79	MBSR + clerkships as usual	Clerkships as usual	BSI	Post-int, 4m, 9m, 12m, 17m	Mindfulness (FFMQ-39), gender, age

See our published AD meta-analysis^20^ for information on the two eligible trials that did not provide individual participant data^62,63^

1m–17m, number of months to follow-up; BSI, Brief Symptom Inventory; CORE-OM, Clinical Outcomes in Routine Evaluation—Outcome Measure; DASS-21, Depression, Anxiety and Stress Scale; FFMQ-x, Five-Facet Mindfulness Questionnaire—x items; HADS-14, Hospital Anxiety and Depression Scale; IM-P, Interpersonal Mindfulness in Parenting Scale; MAAS, Mindful Attention Awareness Scale; *N*, number of studies; OPS, Operational Stress subscale; Post-int, post-intervention; PSS-x, Perceived Stress Scale—x items; SCL-90-R, Symptom Checklist-90-Revised.

Sample sizes for individual trials ranged from 44 to 670 participants. Participant types were diverse, ranging from general university and medical and nursing students to teachers, law enforcement officers and healthcare professionals. In keeping with the inclusion of non-clinical participants, for a cluster RCT of lung cancer patients and partners, only partner data were used^30^, and for one of the parents of children with ADHD, only parent data were used^67^, and only the data from non-asthmatic participants were used in an RCT which also recruited asthmatic participants^65^.

Mean ages reported across studies varied between 19 and 59 years, and females accounted for 11% to 100% of participants across trials. Pooling the IPD observed from all included studies revealed that the median age was 34 (range, 17 to 76; non-normal distribution) and 71% of participants were women. The median number of years of education was 15 (range, 8 to 21; non-normal distribution).

Mean levels of baseline distress and dispositional mindfulness cannot be meaningfully estimated across all studies because different instruments were used to measure them. Considering the most-used psychological distress measure, the 10-item Perceived Stress Scale (6 trials; 1,069 participants, most from the United States), the mean score is 15.48 (s.d., 6.57). This is significantly higher distress (*P* value (*P*) < 0.001) than that of a US probability sample of 2,387 adults with a mean of 13.02 (s.d., 6.35) (ref. ^68^). Scores from 0 to 13 are considered indicative of low stress, whereas those between 14 and 26 are considered indicative of moderate stress^69^. The higher distress level in our sample could be driven by the fact that half of these MBPs are targeted at groups at risk of being distressed. However, reducing the sample to universal (not targeted) MBPs does not substantially change the mean score (15.10; s.d., 6.45). Considering the most-used dispositional mindfulness measure, the 15-item Mindful Attention Awareness Scale (3 trials; 694 participants, most from the United States), the mean score of 4.29 (s.d., 0.75) is not significantly different (*P* = 0.23) from that of a comparable, non-clinical sample of 200 US adults (mean, 4.22; s.d., 0.63) (ref. ^70^). Item-level data for re-calculating total scores were provided for all but two trials, which provided total scores only.

The most-offered MBP was mindfulness-based stress reduction (MBSR). All studies had to include a passive control, but four had additional active-control groups and these included exercise and health enhancement programs and a stress management course.

All the trials measured psychological distress between 1 and 6 months following intervention completion because this was an eligibility criterion. In addition, all the trials measured post-intervention psychological distress (that is, less than 1 month after program completion). Three trials measured psychological distress beyond 6 months after completion of the intervention; the longest follow-up was 17 months post-intervention^71^. Effect modifiers gender and age were available for all trials, education level was available for all but one and dispositional mindfulness was measured in 11 of the 13 included studies.

Trials had on average 20% missing data on the primary outcome, ranging from 1% to 54% (Supplementary Table 1). Half of these missing data (that is, 10% of the total data) came from participants with missing data on all the review outcomes. By arm, 19% of MBP primary outcome data were missing, compared with 22% of passive controls and 16% of active controls.

Communications with collaborating trial authors when risk of bias was unclear led to all trials being classified as low risk for domain 1 (risk of bias arising from the randomization process); before the queries, four trials had been assessed as having some concerns. For the second domain (risk of bias due to deviations from the intended interventions), for the comparisons with passive-control groups, conservatively, all trials remained assessed as having a high risk. For active-controlled comparisons, risk was low. For domain 3 (risk of bias due to missing outcome data), 6 of the 13 trials had initially been assessed as having some concerns, whereas the remainder had been assessed as having a low risk. However, subsequently obtaining IPD for all randomized participants and using prespecified multiple missing data imputation and sensitivity analyses of departures from the missing-at-random assumption resulted in all the trials being reappraised as having a low risk of bias because of missing data. Domain 4 (risk of bias in the measurement of the outcome) remained classified as high risk across all trials as the outcomes were self-reported by nature. For the fifth and last domain (risk of bias in the selection of the reported result), 9 of the 13 trials were originally rated as having some concerns, but as the IPD meta-analysis had prespecified analyses, all trials were then rated as low risk. This resulted in uniform ratings across trials: all trials were low risk for domains 1, 3, and 5 and high risk for domain 4. Domain 2 was high risk for all passive-controlled comparisons and low risk for all active-controlled comparisons (Supplementary Table 2).

### Intervention effects

**Fig. 2:**
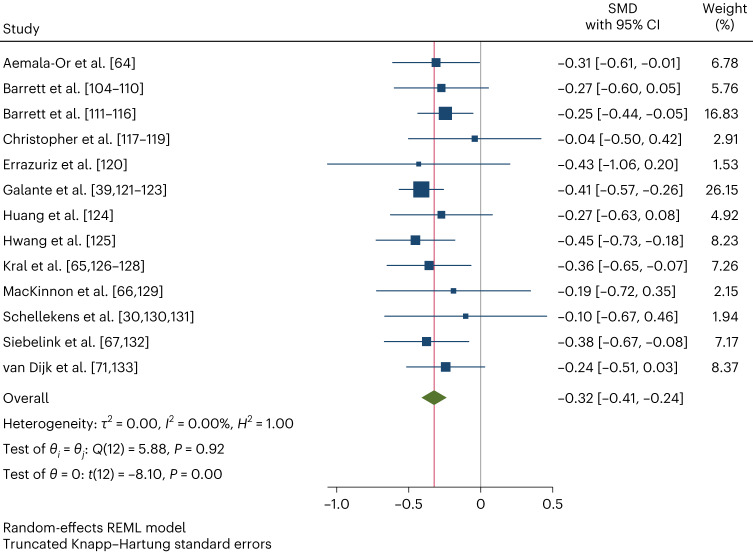
IPD meta-analysis of the primary outcome (psychological distress at the 1–6 month follow-up, comparison with passive-control groups). Random-effects meta-analysis using the restricted maximum-likelihood (REML) method (two-sided test with no adjustment for multiple comparisons). Data are presented as SMD with 95% CIs. *N* = 2,371 participants.

Table 2 shows the results of the IPD meta-analysis assessing the overall effects of MBPs on psychological distress (Fig. 2). In comparison with passive-control groups, on average, MBPs reduce distress between 1 and 6 months post-intervention, our primary outcome (standardized mean difference (SMD), −0.32; 95% confidence interval (CI), −0.41 to −0.24; *P* < 0.001; 95% prediction interval (PI), −0.41 to −0.24 (no heterogeneity)). The effect size, according to Cohen’s conventional criteria^72^, is small to moderate, but the confidence intervals are narrow, and there is no evidence of statistical heterogeneity. Results are similar for the other psychological distress timepoint ranges in comparison with passive-control groups. However, there is no evidence that MBPs decrease psychological distress in comparison with active-control groups.

**Table 2:** Individual participant data meta-analyses comparing the effects of MBPs on psychological distress with control groups, by timepoint range

Control	Time	*N*	SMD	CIL	CIU	*P*	*I*^2^ (%)	PIL	PIU	GRADE confidence
Passive	Post-int	13	−0.28	−0.36	−0.20	0.000	0.00	−0.36	−0.20	Moderate
**Passive**	**1–6m**	**13**	**−0.32**	**−0.41**	**−0.24**	**0.000**	**0.00**	**−0.41**	**−0.24**	**High**
Passive	6+m	13/3^a^	−0.29	−0.40	−0.18	0.000	NA	−0.41	−0.16	Low
Active	Post-int	4	−0.05	−0.35	0.26	0.673	36.19	−0.68	0.59	Very low
Active	1–6m	4	0.03	−0.20	0.25	0.735	0.00	−0.28	0.33	Very low

Random-effects meta-analyses using the restricted maximum-likelihood method (two-sided tests with no adjustment for multiple comparisons). The primary outcome is in bold.

1–6m, follow-up within 1–6 months post-intervention; 6+m, follow-up over 6 months post-intervention; CIL, 95% confidence interval lower; CIU, 95% confidence interval upper; NA, not applicable; PIL, prediction interval lower; PIU, prediction interval upper.

^a^Multivariate meta-analysis: number of studies that contributed to the result/number of studies that measured the outcome.

**Fig. 3:**
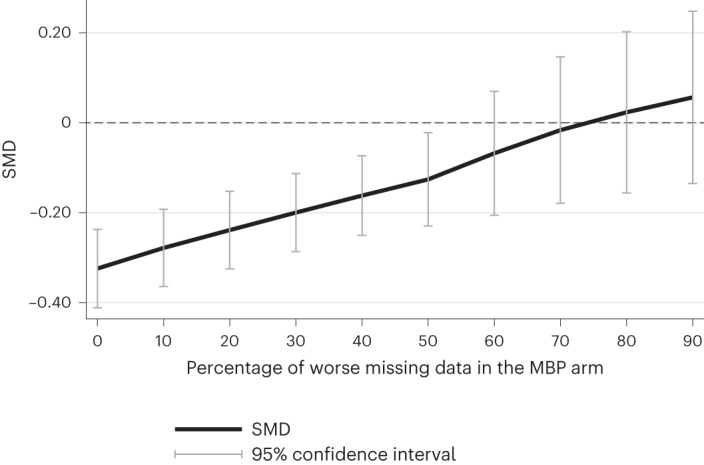
Sensitivity analysis exploring non-missing-at-random intervention data scenario. *N* = 2,371 participants.

Incorporating published data from the two trials for which IPD were not available did not modify the primary outcome’s effect estimate size or significance (15 trials; 2,477 participants; SMD, −0.31; 95% CI, −0.40 to −0.23; *P* < 0.001; *I*² index (*I*^2^), 0%). Similar results were obtained by analyzing observed data only (13 trials; SMD, −0.32; 95% CI, −0.41 to −0.23; *P* < 0.001; *I*^2^, 0%) and by modeling missing data as 10% or 20% worse than the observed data (for both scenarios: 13 trials; SMD, −0.32; 95% CI, −0.40 to −0.23; *P* < 0.001; *I*^2^, 0%). Figure 3 shows that missing outcome scores in the MBP arm would need to be over 50% worse on average than the observed scores to impact the statistical significance of reported effects, and over 70% worse for the direction of the intervention effect to change.

As a further check, we compared the primary outcome AD and IPD meta-analysis results of the nine trials that overlap in this publication and in our previous review^20^. Our previous review included 27 trials for this IPD meta-analysis’s primary outcome, and found results similar to those of the IPD meta-analysis, but with much greater heterogeneity. By comparing the same set of nine trials, we aimed to explore whether the heterogeneity could be explained by the assumptions and transformations needed when extracting summary data from publications in aggregate-data meta-analyses as opposed to using IPD. Effect sizes were similar, but the AD meta-analysis was more heterogeneous (SMD, −0.28; 95% CI, −0.44 to −0.12; *P* = 0.004; *I*^2^, 40%; 95% PI, −0.62 to 0.07) than the IPD meta-analysis (SMD, −0.32; 95% CI, −0.42 to −0.22; *P* < 0.001; *I*^2^, 0%).

According to the Grading of Recommendations Assessment, Development and Evaluation (GRADE) assessment (Table 2 and more details in Supplementary Table 3), confidence in the results emerging from 1 to 6 month follow-up comparisons with passive-control groups (primary outcome) is high. Confidence in the post-intervention results is moderate because of non-reporting bias potentially arising from the exclusion of trials that only reported secondary outcomes. Confidence in the 6+ month follow-up results is low because of non-reporting bias and imprecision (small number of studies), and confidence in the results arising from comparisons with active controls is very low because of non-reporting bias, imprecision, and inconsistency (unexplained heterogeneity).

### Effect modifiers

Table 3 summarizes the interaction effects for all outcomes and comparisons. We found no evidence that any prespecified variables modified the effects of MBPs on our primary outcome, psychological distress at 1–6 months post-intervention (Supplementary Figs. 1–5), or any of our secondary outcomes. Because none of the prespecified candidate variables showed evidence of interaction effects, we were unable to build a predictive model to show which profiles could benefit the most.

**Table 3:** Individual participant data interaction meta-analyses by control group and timepoint range

Control	Time	Modifier^a^	*N*	Mod SMD	CIL	CIU	*P*	*I*^2^ (%)	PIL	PIU
Passive	Post-int	Age	13	0.00	−0.01	0.01	0.863	0.69	−0.01	0.01
		Gender	10	0.01	−0.19	0.22	0.890	0.26	−0.20	0.22
		Distress	13	−0.07	−0.15	0.01	0.089	0.00	−0.15	0.01
		Education	11	−0.03	−0.07	0.02	0.239	2.34	−0.08	0.03
		Mindfulness	11	0.03	−0.07	0.13	0.497	0.00	−0.07	0.13
**Passive**	**1–6m**	**Age**	**13**	**−0.01**	**−0.02**	**0.01**	**0.417**	**19.45**	**−0.03**	**0.02**
		**Gender**	**11**	**0.11**	**−0.13**	**0.36**	**0.330**	**14.97**	**−0.29**	**0.52**
		**Distress**	**13**	**−0.06**	**−0.15**	**0.03**	**0.193**	**0.00**	**−0.15**	**0.03**
		**Education**^**b**^	**11**	**−0.01**	**−0.05**	**0.03**	**0.586**	**0.00**	–	–
		**Mindfulness**	**11**	**0.05**	**−0.06**	**0.17**	**0.314**	**0.00**	**−0.06**	**0.17**
Passive	6+m	Age	13/3^c^	−0.01	−0.02	0.01	0.344	NA	−0.02	0.01
		Gender	11/3^c^	−0.01	−0.29	0.26	0.925	NA	−0.37	0.35
		Distress	13/3^c^	−0.05	−0.16	0.06	0.393	NA	−0.17	0.08
		Education	11/2^c^	0.05	−0.02	0.13	0.188	NA	−0.07	0.17
		Mindfulness	11/2^c^	−0.02	−0.20	0.17	0.864	NA	−0.25	0.25
Active	Post-int	Age	4	0.01	−0.01	0.03	0.301	0.04	−0.02	0.03
		Gender	2	0.21	−2.00	2.41	0.445	0.00	–	–
		Distress	4	−0.02	−0.23	0.19	0.802	0.00	−0.30	0.26
		Education	4	0.04	−0.09	0.16	0.399	17.71	−0.19	0.26
		Mindfulness	4	0.11	−0.10	0.32	0.189	0.00	−0.17	0.40
Active	1–6m	Age	4	0.01	−0.01	0.03	0.209	0.03	−0.02	0.04
		Gender	2	0.12	−2.19	2.44	0.619	0.00	–	–
		Distress	4	0.22	−0.33	0.76	0.298	80.42	−1.28	1.71
		Education	4	−0.01	−0.17	0.14	0.799	37.39	−0.35	0.32
		Mindfulness	4	−0.02	−0.46	0.41	0.872	62.84	−1.09	1.04

Random-effects meta-analyses using the restricted maximum-likelihood method (two-sided tests with no adjustment for multiple comparisons). Primary outcome in bold.

Mod SMD, effect-modifier SMD, which is the change in SMD per one unit change in effect modifier.

^a^Measurement units are years for age and education and standard deviations for distress and mindfulness. The base gender is male.

^b^The random-effects model did not converge, so the fixed-effects model is reported.

^c^Multivariate meta-analysis: number of studies contributing to the result/number of studies that measured this outcome.

Gender-specific meta-analyses suggest that MBPs reduce distress among both men and women compared with passive controls (for men: SMD, −0.40; 95% CI, −0.55 to −0.25; *P* < 0.001; for women: SMD, −0.28; 95% CI, −0.38 to −0.18; *P* < 0.001). Two trials in the primary outcome meta-analysis used student samples. Whereas students are projected to gain more years of education as they progress in their studies, participants selected from the community with the same number of years of education are less likely to gain additional years of education in the future. Thus, although all the interaction models were adjusted by age, we ran a post hoc sensitivity analysis excluding student trials to explore the possibility that students confounded education–MBP interactions. We found no interaction effect difference between this restricted analysis (SMD, 0.02; 95% CI, −0.03 to 0.08; *P* < 0.40) and that which includes all studies.

## Discussion

Our IPD meta-analysis found evidence that MBPs generate, on average, a small to moderate reduction in adults’ psychological distress, lasting for at least 6 months in each of the represented settings, in comparison with no intervention. Based on the GRADE assessment, our confidence in these results is high. There was no clear indication that this effect is modified by baseline psychological distress, age, gender, education level or dispositional mindfulness.

### Intervention effects

Our intervention effect results encourage the implementation of teacher-led MBPs for adults in non-clinical settings, and we have not found subgroups or settings in which they may be less efficacious. However, the average effect is small to moderate, and it is difficult to ascertain clinical significance because we have combined different instruments, although the effect size is within the range that has been proposed for defining minimally important difference based on effect size^73^. Also, our intervention effect results only estimate average effects across participants. There is evidence that some people may not benefit or may even experience harm^74^.

Despite our primary outcome results being positive, without active comparisons or blinded controls, we cannot confidently say that this is due to mindfulness training. Furthermore, in our exploratory analysis, we found no clear evidence that MBPs are superior to other interventions in mental health promotion. These results are aligned with those of our AD meta-analysis, which included up to 51 active-controlled trials^20^, and a meta-review of MBP meta-analyses^22^. Therefore, the specificity of MBP effects is still unclear.

Our findings do not extend to automated or self-guided MBPs such as those delivered through smartphone applications, books, CDs, etc. The lack of human interaction and teacher guidance may substantially modify their effectiveness and safety^75^. Despite the popularity of app-based mindfulness courses, potentially driven by cost and accessibility advantages^14,76^, the evidence base is still developing^77^. Our findings may partially extend to teleconference-based MBPs, but implementation research should better define this.

Similarly, our findings are limited to voluntary MBPs and should not be automatically extended to other types of offerings. The evidence so far on compulsory MBPs for adults, for example, an MBP required as part of medical school courses, is scarce and does not show benefit^78^. Furthermore, a recent large and well-conducted trial assessing a non-clinical MBP for adolescents, delivered as part of the school curriculum, showed no added benefit to well-being compared with normal school provision^79^.

### Effect modifiers

Our findings on effect modification are remarkably similar to those of our IPD meta-analysis of MBCT to prevent recurrent depression relapse: no evidence was found of the effect-modifying role of age, sex, education or dispositional mindfulness on MBP effects^34^. However, the depression relapse IPD meta-analysis found some evidence suggesting a relative increment in effect with worse baseline mental health status, which was not replicated in the current non-clinical study.

Other study designs, mainly individual trials and AD meta-analyses, have found different results for all of these potential effect modifiers, for example, ^26,27,36,38–40^. However, study designs other than the IPD meta-analysis suffer from methodological shortcomings that make interaction analyses of potential effect modifiers at the individual participant level less reliable^80,81^.

Our results may help in the interpretation of evidence that MBPs targeted at at-risk or stressed groups are more effective in reducing depression and anxiety than universal MBPs. Our AD meta-analysis found that indicated MBPs (for individuals with subclinical symptoms of mental health conditions) and selective MBPs (for those at higher risk of developing mental health problems, such as carers) were more beneficial in preventing anxiety and depression than universal MBPs, although no such differences were found for psychological distress^20^. Another AD meta-analysis found similar results for depressive symptoms among university students^82^. Our current IPD meta-analysis suggests that this greater effect of targeted MBPs is not related to those more distressed at baseline obtaining more benefits but could instead be due to differences in the types of MBPs or their teachers (for example, therapists teaching selective or indicated MBPs). Another explanation could be that depression and anxiety questionnaires may suffer from ceiling effects among those less distressed, of which there are more in universal interventions, whereas psychological distress questionnaires may retain sensitivity along this mental health spectrum. Alternatively, those with higher baseline distress may be more responsive to MBP effects, but they may also have less time to devote to mindfulness practice after the course, so the extra effects may not materialize after the course ends.

Individuals with fewer than 12 years of education were underrepresented in our dataset, so we cannot exclude the possibility that benefit differently from MBPs. Our sample reflects the wider situation: the average rates of educational attainment for the individuals in MBSR and MBCT trials tend to be greater than the average for the US population^83,84^. Individuals with low educational attainment may not have equal access to MBPs. This could happen at least in part because of the aforementioned offering of MBPs by universities and workplaces, granting those enrolled in higher-degree programs and working for well-resourced employers access to MBPs while leaving the wider public with limited access. MBPs available to the wider public may be less common than MBPs behind the paywall of being enrolled in higher-degree programs or employed by well-resourced employers. Our results warrant efforts to adapt and thoroughly evaluate MBPs for wider audiences.

Our IPD has a good age range among adults, although those above 70 years old were underrepresented, so our findings cannot reliably extend to them. Men were underrepresented, making up 29% of the participants, which reflects other MBP trials very precisely^83,84^. Given that we found MBPs to be effective among men, more research is needed to identify barriers to access or engagement among men. Factors such as the gender of teachers and peers may contribute to engagement; in the case of MBPs within trials, the attractiveness of the control condition (for example, physical exercise) or researchers’ characteristics may play roles.

A key limitation of IPD meta-analyses is that the effect modifiers that can be assessed are limited to those that the existing trials measured. Other effect modifiers may be at play, such as participant expectations and beliefs, group and setting dynamics, and personality and cognitive factors^28^. Socio-economic and cultural factors may also be key. Although our country spread was good, low- and middle-income countries are underrepresented, and low- and middle-income populations within countries may be underrepresented too. Future qualitative research could identify and prioritize the most promising potential effect modifiers^85^.

In order to reduce the likelihood of spurious effect modification results, we had to limit our analyses to a handful of potential effect modifiers, and we could not assess any nuances within these (for example, different dimensions of baseline psychological distress). Also, our assessed effect modifiers may have shown significant results with more trials included. We hope that these limitations are addressed in future IPD meta-analyses as trials in the field continue to accumulate.

### Confidence in the results

This IPD meta-analysis was preceded by a comprehensive systematic review, the methods were prespecified, and various risks of bias were mitigated. These aspects increased the robustness of our results^86^. We were able to obtain IPD for 96% of the eligible participants, over the 90% mark, which is seen as a reliable indicator of low risk of selection bias^86,87^. In line with recent work^88^, results were robust to sensitivity analyses including AD for the two eligible trials from which we could not obtain IPD.

Confidence in the IPD meta-analysis intervention effect primary outcome result is high according to the GRADE assessment, meaning that further research is unlikely to change this result. The assessment is markedly higher than that of our AD meta-analysis^20^. Several aspects explain this difference. In contrast to the AD meta-analysis, the trials included in the IPD meta-analysis passed an a priori quality threshold. Limiting inclusion to those trials with higher quality can make a meta-analysis more robust^87^. Many of our AD meta-analysis results were sensitive to trial quality, which encouraged us to limit our IPD meta-analysis. We acknowledge, however, that the revised Cochrane Risk-of-Bias tool (RoB 2) has not been validated as a scale, so we could not use validated cut-off points for selecting trials and risk-of-bias domains may not be interchangeable^89^. All trials’ risk of bias was reduced further by using IPD.

The consistency of the results also contributed to the GRADE rating. AD meta-analyses rely on published data, which limits the ability to check it and forces analysts to make transformations and strong assumptions of a varied, complex and hard-to-prespecify nature. These AD meta-analysis limitations increase the chance of biases and errors, decreasing the reliability of results and sometimes inflating heterogeneity. The differences between our AD and IPD meta-analyses illustrate this. The narrow prediction intervals of our primary outcome contrast with those of our previous AD meta-analysis. In the latter, we found similar results, but given the heterogeneity between studies, the findings did not support the generalization of MBP effects across every represented setting^20^. The study-level moderators that we investigated in the AD meta-analysis, such as program characteristics or type of population targeted, were not able to fully explain the observed heterogeneity. Similarly, we have not found strong evidence of individual-level effect modifiers in the IPD meta-analysis reported here. Instead, methodological factors may have contributed to the increased precision of both confidence and prediction intervals in the IPD meta-analysis compared with the AD meta-analysis, significantly reducing heterogeneity.

The methodological aspects of our study described above illustrate many of the advantages of IPD meta-analyses over AD meta-analyses. Specifically, they show the strengths of using IPD to re-calculate summary measures and of applying the same summary measures, sample types, and imputation methods to all the trials. The gains in consistency and reliability tend to compensate for the extra time and resource involved in collecting IPD, which usually limits the number of studies that can be included in IPD meta-analyses in comparison with their AD counterparts. Contemporary open-data initiatives mean that increasing amounts of IPD will be readily available from public data repositories, which will undoubtedly make IPD meta-analyses more feasible and faster over time.

However, despite the use of IPD, there are remaining risk-of-bias concerns regarding the lack of blinding and self-reported outcomes. These are inherent to the nature of the research field: it is extremely difficult to effectively blind participants to real-life psychosocial interventions, and psychological distress is an inherently subjective outcome. Future research could consider alternative assessment approaches, such as clinician-rated or partner-rated measures of psychological distress. In addition, downstream effects of psychological distress, such as work absenteeism or health problems, could be considered. Future trials should also take into account RoB 2 domains and trial reporting checklists when planning their trials in order to increase quality and thereby confidence in their results. Publicly registering a trial protocol ahead of data collection that pre-specifies a primary outcome measure and a primary timepoint, with a primary outcome data analysis plan, could go a long way in this sense. Missing data problems would be greatly mitigated if trialists could encourage participants to complete outcome surveys even when they abandon the MBP.

Confidence in results arising from actively controlled comparisons is very low. These comparisons included very few studies, so results are unreliable. Also, our search excluded studies in which the only comparisons tested were those with active-control groups, potentially biasing the results for this comparison. In any case, it is hard to interpret the effects of MBPs using comparisons with a mix of active-control groups because different control interventions may have different specific effects that may overlap differently with those of the MBPs. Similarly, the effect modification results for this comparison are hard to interpret because the effects of each active-control intervention may be modified in different ways. Confidence will increase as new trials in the field accumulate and we are able to synthesize evidence comparing MBPs with specific interventions, rather than with a mix of interventions.

Time and resource constraints meant that we had to exclude several trials that were only reporting secondary outcome results. We report these secondary outcomes anyway for completeness and for exploratory purposes, but we acknowledge that the limited inclusion could have biased these results, hence the reduced GRADE confidence in them. Relatedly, defining the primary outcome timepoint range as between 1 and 6 months post-intervention and choosing the longest follow-up within this window in order to focus on effects that are widely reported yet likely to be more stable than immediate effects were reasoned and predefined decisions, but ultimately arbitrary.

## Conclusion

In contrast to those who take no action, community adults who choose to take part in group-based, teacher-led MBPs will generally experience a reduction in their psychological distress. Based on the trials accrued so far, we found no clear indication that baseline distress, gender, age, education level or dispositional mindfulness will modify this effect, but further research on MBP effect modification factors is needed.

## Methods

The protocol for this work was prospectively registered (PROSPERO registration number CRD42020200117) and published^90^. Methodological details can be found in the protocol publication and are briefly described below. This study is reported in accordance with the relevant PRISMA guidelines^91,92^. This publication has considered the Global Code of Conduct, a code of ethics for equitable research partnerships. Because this project only involved the use of secondary anonymized data from other research studies, it did not require ethics committee approval.

A public stakeholder group has provided input throughout the life of this project, initially by providing feedback on the study protocol and screening and extracting records as research partners. Then, they contributed to the interpretation, dissemination, and output of the study findings, co-creating a film summarizing the study methodology and key findings and co-authoring a paper (in preparation) detailing their experience in acting as stakeholders on an IPD meta-analysis project. We have also involved a group of professional stakeholders to form an advisory group.

### Study search and selection

The search for eligible studies follows the same protocol conducted as part of our previous review^20^, wherein 13 databases (AMED, ASSIA, CENTRAL, CINAHL, ERIC, EThOS, EMBASE, MEDLINE, ProQuest, PsycINFO, Scopus, Web of Science and the World Health Organization International Clinical Trials Registry Platform) were electronically searched with no publication date limits. We updated this search in December 2020 using the search strategies prespecified in our IPD meta-analysis protocol^90^. In addition to studies obtained through database searches, the 136 trials included in our previous review were screened for eligibility against the IPD meta-analysis inclusion criteria. Moreover, authors invited to share IPD made us aware of further publications linked to their main trial publications.

The review inclusion criteria, applied at the study level, were (1) parallel-arm RCTs, including cluster RCTs; (2) group-based first-generation MBPs^19^, with a minimum intensity of four 1 h in-person teacher-led sessions or equivalent; (3) passive-control groups, such as no intervention or waitlists, or treatment as usual, which the MBP arm also had to have access to; (4) adult (18+ year old) participants living in the community who were not specifically selected for having any particular clinical condition; (5) self-reported psychological distress measured between 1 and 6 months after MBP completion; (6) at least one of the following candidate effect modifiers being reported: baseline psychological distress, gender, age, education and dispositional mindfulness; and (7) a maximum of two risk-of-bias sources rated as ‘high’, before having obtained IPD, according to RoB 2 (ref.^93^).

Our inclusion criteria were deliberately narrower in scope than our previous AD meta-analysis review criteria, rendering our IPD meta-analysis feasible and allowing for a more focused and high-quality analysis. For feasibility purposes, we had to limit our IPD meta-analysis inclusion criteria to those trials reporting the primary outcome, excluding those trials only reporting secondary outcomes. We have also excluded trials which only compared MBPs with active-control groups, but we did include trials that had active-control groups if they also compared MBPs with a passive-control group.

Retrieved records (first, abstracts, then full texts) were screened independently by two reviewers (J.G. and C.F.) using Covidence^94^ for all but criterion (7). Then, multiple records from the same trial were combined and appraised using RoB 2 (7). Any discrepancies in screening or rating decisions were discussed and resolved within the research team.

### Data collection and processing

Two independent reviewers (J.G. and C.F.) extracted study-level characteristics from publications. We contacted the authors of eligible trials and invited them to collaborate, offering co-authorship on any publications resulting from their shared trial data. Initial contact by email included the review protocol and instructions on which data were being requested and how to transfer the data. We requested final and baseline scores for the outcome measure and the available prespecified effect modifiers. Where trials used more than one measure for the same outcome, we requested the more psychometrically robust outcome. Where trials measured the same outcome multiple times within our timepoint range of interest (for example, the 2 and 4 month follow-up), we requested the longest follow-up to reduce heterogeneity within the range, as well as to focus on the effects that are likely to be more stable.

We asked authors to share anonymized IPD for all randomized participants, including any data which may have been omitted from trial publications or analyses. We requested data for individual scale items rather than calculated total scores, without imputation of missing data. Data transfers were completed using the University of Cambridge Transfer Server of the Secure Data Hosting Service, an ISO 27001-certified safe haven for sensitive data. When transfers were finished, one reviewer (C.F.) checked all trial data files with guidance from a second reviewer (J.G.).

We initially checked files in the original format they were sent in (SPSS, Excel) and again after importing into Stata. First, we confirmed that all randomized participants were present by checking trial publications and registries. Second, we checked all files for any missing variables. Third, we checked that scores were for individual scale items, not total scores, and whether these were reversed or imputed, whether data were missing and how this was indicated in the original file, and whether there were any extreme values or inconsistent items (for example, unusually old or young participants). Where authors also transferred a data dictionary, we compared it with the data files and standardized it across studies. Where necessary, we contacted authors when we had questions or needed clarifications and we discussed with two reviewers (J.G. and C.F.) and recorded any changes to the original data files, such as the removal of ineligible participants. Once we completed checking all trial data files, we standardized them into a prespecified format using Stata.

Once they were standardized, we calculated the demographics and descriptive statistics of each trial and timepoint and compared them with those of trial publications; we also discussed any discrepancies, and if they remained unresolved, we contacted the trial authors for further information. Then, we conducted analyses of individual trials and compared them with published analyses, discussing any discrepancies with the trial authors. For studies in which IPD were not available, two independent reviewers extracted AD from trial publications. Trials used different categories for collecting education-level data, so we estimated years of education based on the education systems of the countries in which the trial was conducted.

### Outcomes and risk-of-bias assessment

The primary outcome of this meta-analysis is self-reported psychological distress measured between 1 and 6 months after program completion using psychometrically valid questionnaires (for example, Perceived Stress Scale, General Health Questionnaire, Depression, Anxiety and Stress Scale). Psychological distress was the most measured and robust outcome of our AD meta-analysis^20^ and is normally distributed in the general population. Rather than focusing on any particular set of mental health symptoms, psychological distress transcends the diagnostic categories traditionally used in psychiatry and is a general indicator of mental health deterioration^9^.

Secondary outcomes are psychological distress measures taken less than 1 month after program completion (post-intervention) or beyond 6 months after program completion. Post-intervention timepoints were considered to be secondary outcomes because they do not inform stable changes; therefore, they are less useful for understanding the real-life impact of MBPs and the factors modifying these effects in a stable fashion. Secondary outcome analyses were considered exploratory.

The reviewers selecting the studies independently assessed whether the trial outcomes included measures of psychological distress. Disagreements were resolved via consensus between two senior team members (T.D. and P.B.J.) blinded to the trial results and to which trial used each measure, before IPD were requested. Measure validity was ascertained by cross-referencing the validity and reliability studies cited in the trial publications. Where measures had been translated from the original language, validity studies of the translated measures were additionally checked. As trials used different instruments to measure psychological distress, we standardized them using *z* scores.

The main analysis compared MBPs with a combination of all the passive-control groups. We chose this as the main comparison because it makes the interpretation of potential modification effects more straightforward. Including active controls would make results hard to interpret because the effects of each control intervention may be modified in different ways. A comparison with passive-control groups allows for a better understanding of MBP effect modification per se. Notwithstanding, if the included trials also compared MBPs with other interventions, these were grouped under the comparator ‘active control’ for exploratory analyses.

Relying initially on trial publications, two reviewers (J.G. and C.F.) independently assessed trials’ risk of bias using RoB 2 for RCTs applied to the review outcomes^93,95^. This tool considers bias due to (1) randomization, (2) deviations from intended interventions, (3) missing outcome data, (4) measurement of the outcome and (5) selection of the reported result. We assessed cluster RCTs with their specific sub-set of questions on RoB 2. Once IPD had been obtained, we updated the risk-of-bias assessments for individual studies (for example, the risk was lowered if IPD included participants missing in published trial reports) and discussed any unclear information with study authors. Finally, we used the GRADE approach to assess the confidence in the accumulated evidence^96^. It categorizes the quality of evidence into four levels of certainty: high, moderate, low, and very low. For each outcome, we considered trials’ risk of bias, meta-analysis non-reporting bias, imprecision (CIs), inconsistency (prediction intervals) and indirectness of evidence.

### Analytic approach

To calculate the overall MBP effect, we performed two-stage IPD meta-analyses^54^. Meta-analyses were univariate for the timepoint ranges for which data from all the trials were available; otherwise, they were multivariate, including all available timepoint ranges to make the most efficient use of available data^97^.

For the first stage of each IPD meta-analysis, we conducted linear regressions separately by trial to estimate the intervention effects following the intention-to-treat principle. We used the analysis of covariance estimate as an effect measure (final score adjusted for baseline score and the available prespecified effect modifiers)^54,98^. We treated questionnaire scores as continuous variables.

For the second stage of the IPD meta-analyses, we combined the intervention effects from each trial using pairwise random-effects meta-analyses within comparator categories. We used restricted maximum likelihood to estimate heterogeneity in the intervention effect and quantified heterogeneity using approximate prediction intervals^99^.

We imputed missing data following a prespecified plan^54,100,101^. Multiple imputation (multivariate imputation by chained equations) was performed separately by trial and by randomized group within each trial. We only imputed data for participants for which data on other review outcomes were available, and we used these other outcomes as auxiliary variables (except in the sensitivity analyses assessing departures from the missing-at-random assumption, in which we imputed all missing outcome data; see below). In order not to increase between-study heterogeneity, we used the same set of covariates in the imputation models across studies^54^: the psychological distress outcomes and prespecified effect modifiers measured by that study. We performed 50 imputations per study, which was at least equal to the percentage of incomplete cases. More details are found in our protocol^90^.

We performed a series of sensitivity analyses exploring missing data for the primary outcome. One analysis incorporated published AD from trials for which IPD were unavailable into the second stage of the two-stage IPD meta-analysis^60^. Another sensitivity analysis was performed using no imputed data. We assessed departures from the missing-at-random assumption in sensitivity analyses by increasing all imputed psychological distress scores by 10% and 20% and by increasing them by 10–90% in the intervention arm only, given that MBP participants experiencing deterioration may have been less willing to complete outcome measures than passive-control-group participants, who may have expected to feel worse.

The effect modification analyses assessed the potential modifiers of interest one by one following a within-studies approach^80,102^. For each effect modifier, a treatment-by-participant covariate interaction term was incorporated in the intervention effect trial regression models. Then, the estimated interactions were combined in a random-effects meta-analysis. We estimated subgroup-specific intervention effects by repeating the analysis procedure using the interaction parameters derived from the within-studies approach.

### Reporting summary

Further information on research design is available in the Nature Portfolio Reporting Summary linked to this article.

### Supplementary information

Supplementary tables and figures.

Reporting Summary

Supplementary Data PRISMA checklist

## Data Availability

The aggregate data are publicly available^103^. For individual trial data availability, please refer to the relevant trial publication.

## Code availability

The statistical code is publicly available^103^.
