# Supplementary material for: Systematic review and individual participant data meta-analysis of randomized controlled trials assessing mindfulness-based programs for mental health promotion
Source: Nat Ment Health. 2023 Jul 10;1(7):462–76. doi: 10.1038/s44220-023-00081-5 (PMC7615230; doi:10.1038/s44220-023-00081-5)
Supplement: Supplementary file 1 [file 44220_2023_81_MOESM1_ESM.pdf]

nature mental health

Analysis

https://doi.org/10.1038/s44220-023-00081-5

Systematic review and individual participant data meta-analysis of randomized controlled trials assessing mindfulness-based programs for mental health promotionIn the format provided by the authors and unedited

Individual participant data systematic review and meta-analysis of randomised controlled trials assessing adult mindfulness-based programmes for mental health promotion in non-clinical settings

Supplementary materials

Table of Contents

Supplementary Tables 2
Supplementary Figures 5

Supplementary Tables

Table 1. Percentage missing data on primary outcome

Dataset		Missing (%)
All		20
By Arm	MBP	19
	Passive	22
	Active	16
By Study	Aeamla	1
	Barrett2012	4
	Barrett2018	7
	Christopher	20
	Errazuriz	54
	Galante	24
	Huang	30
	Hwang	33
	Kral	22
	MacKinnon	30
	Schellekens	30
	Siebelink	10
	vanDijk	22

Table 2 . Risk of bias assessment for individual studies.

Study	D1	D2	D3	D4	D5
Aeamla-Or 2015	Low	High	Low	High	Low
Barrett 2012	Low	High for Passive Control. Low for Active Control.	Low	High	Low
Barrett 2018	Low	High for Passive Control. Low for Active Control.	Low	High	Low
Christopher 2018	Low	High	Low	High	Low
Errazuriz 2020	Low	High for Passive Control. Low for Active Control.	Low	High	Low
Galante 2018	Low	High	Low	High	Low
Huang 2015	Low	High	Low	High	Low
Hwang 2019*	Low	High	Low	High	Low
Kral 2019	Low	High for Passive Control. Low for Active Control.	Low	High	Low
MacKinnon 2021	Low	High	Low	High	Low
Schellekens 2017	Low	High	Low	High	Low
Siebelink 2021*	Low	High	Low	High	Low
Van Dijk 2017*	Low	High	Low	High	Low

The RoB2 tool measures potential bias across five sources (called ‘domains’ in the tool): D1 (randomisation); D2 (deviations from intended interventions); D3 (missing outcome data); D4 (measurement of the outcome); and D5 (selection of the reported result). * Cluster RCTs, which were assessed with their specific sub-set of questions. Abbreviations: high (high risk); low (low risk).

Table 3. Grading of Recommendations Assessment, Development and Evaluation (GRADE) assessment details by confidence domain: risk of bias, non-reporting bias, imprecision, inconsistency, and indirectness.

Control group	Time point	RoB	Non-rep bias	Imprecis ion	Inconsiste ncy	Indirect ness	GRADE confidence
passive	post-int	Not serious	Serious	Not serious	Not serious	Not serious	Moderate
passive	1-6m	Not serious	Not serious	Not serious	Not serious	Not serious	High
passive	6+m	Not serious	Serious	Serious	Not serious	Not serious	Low
active	post-int	Not serious	Serious	Serious	Serious	Not serious	Very Low
active	1-6m	Not serious	Serious	Serious	Serious	Not serious	Very Low

Abbreviations: RoB (Risk of bias); Post-int (follow-up at post-intervention); 1-6m (follow-up within 1-6 months post-intervention); 6+m (follow-up over 6 months post-intervention).

Supplementary Figures

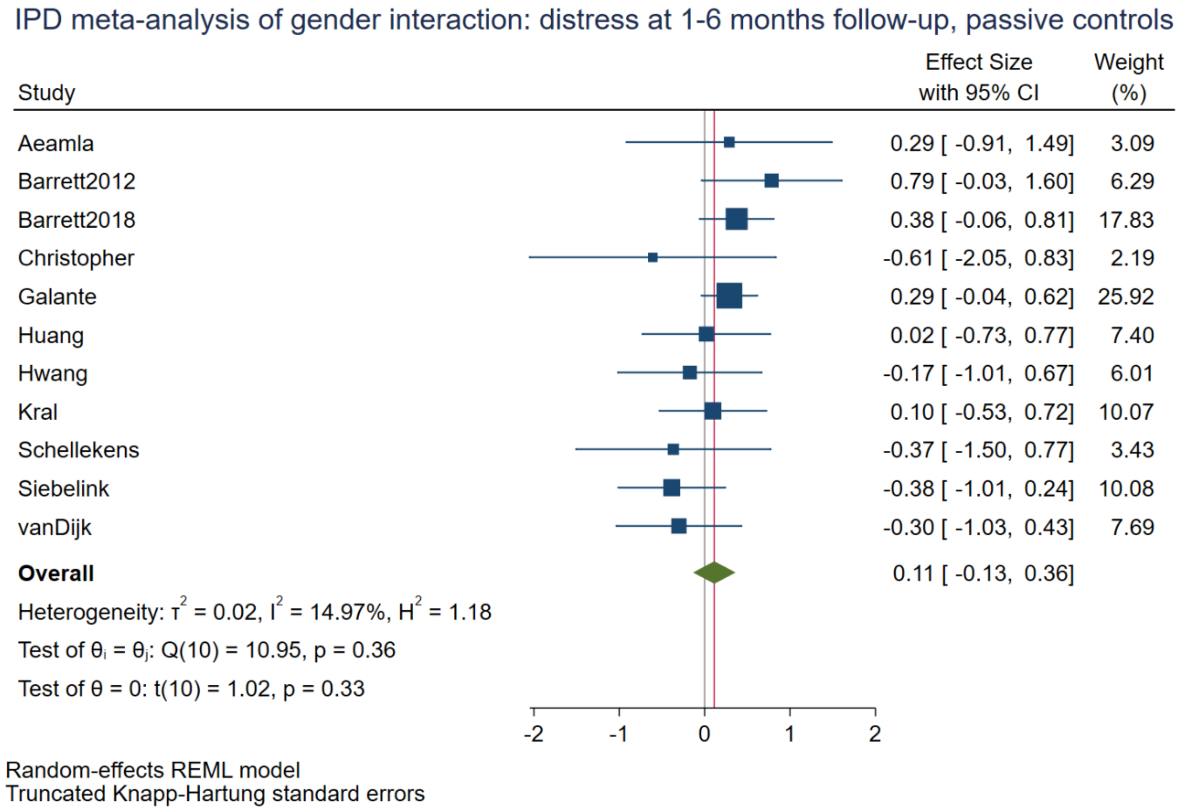

Supplementary Figure 1. Individual participant meta-analysis of gender interaction: distress at 1-6 months follow-up, passive controls. Random-effects meta-analysis using the restricted maximum likelihood method (two-sided test with no adjustment for multiple comparisons). N= 2,206 participants. Data are presented as standardised mean differences (SMD) with 95% confidence intervals (CI).

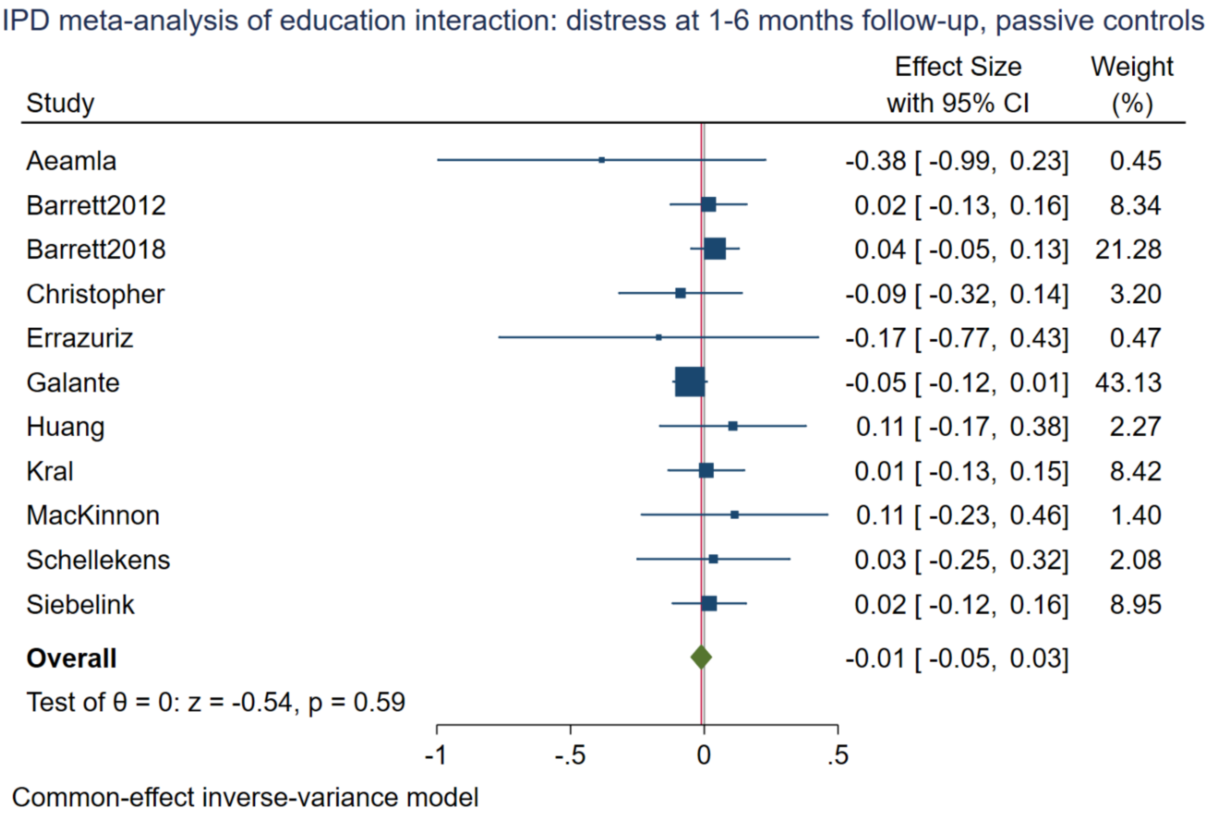

Supplementary Figure 2. Individual participant meta-analysis of education interaction: distress at 1-6 months follow-up, passive controls. Random-effects meta-analysis using the restricted maximum likelihood method (two-sided test with no adjustment for multiple comparisons). N= 2,019 participants. Data are presented as standardised mean differences (SMD) with 95% confidence intervals (CI).

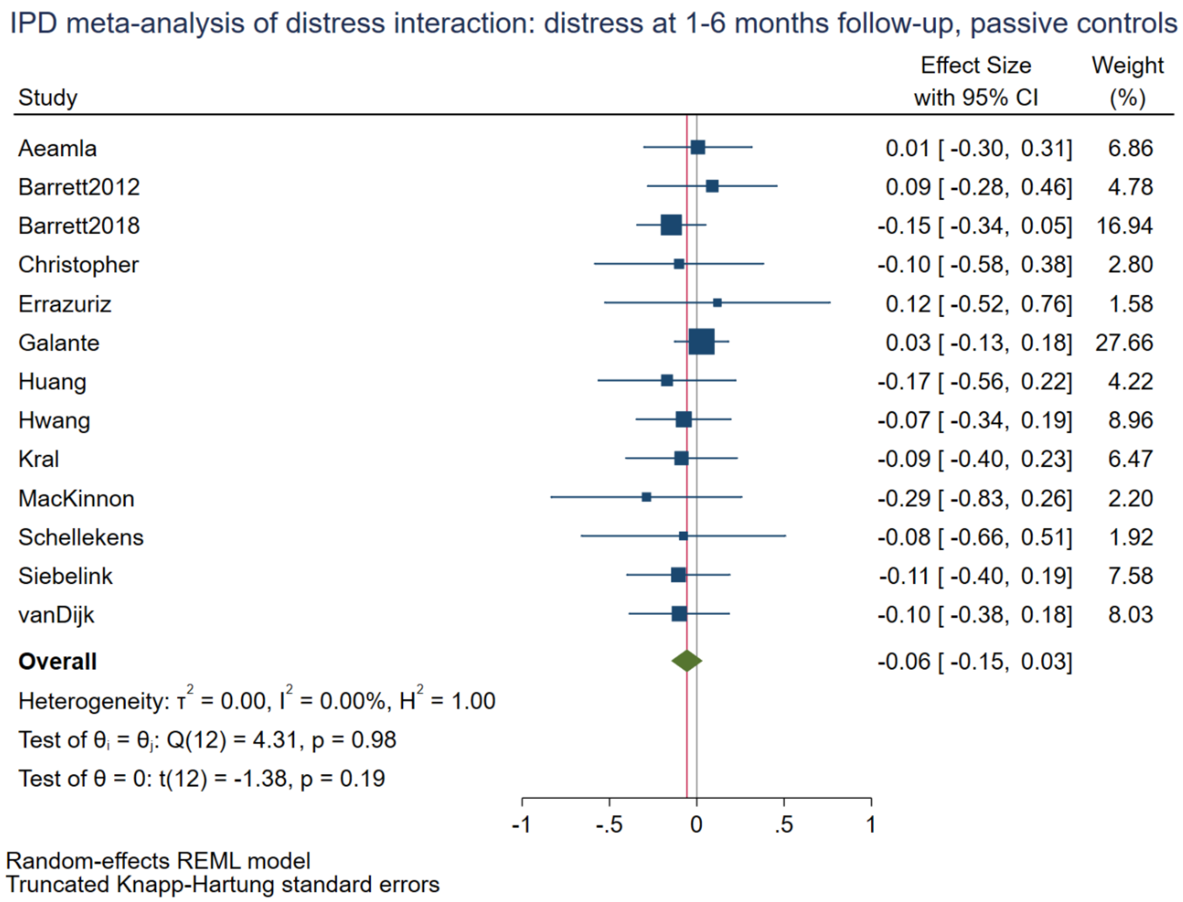

Supplementary Figure 3. Individual participant meta-analysis of distress interaction: distress at 1-6 months follow-up, passive controls. Random-effects meta-analysis using the restricted maximum likelihood method (two-sided test with no adjustment for multiple comparisons). N= 2,371 participants. Data are presented as standardised mean differences (SMD) with 95% confidence intervals (CI).

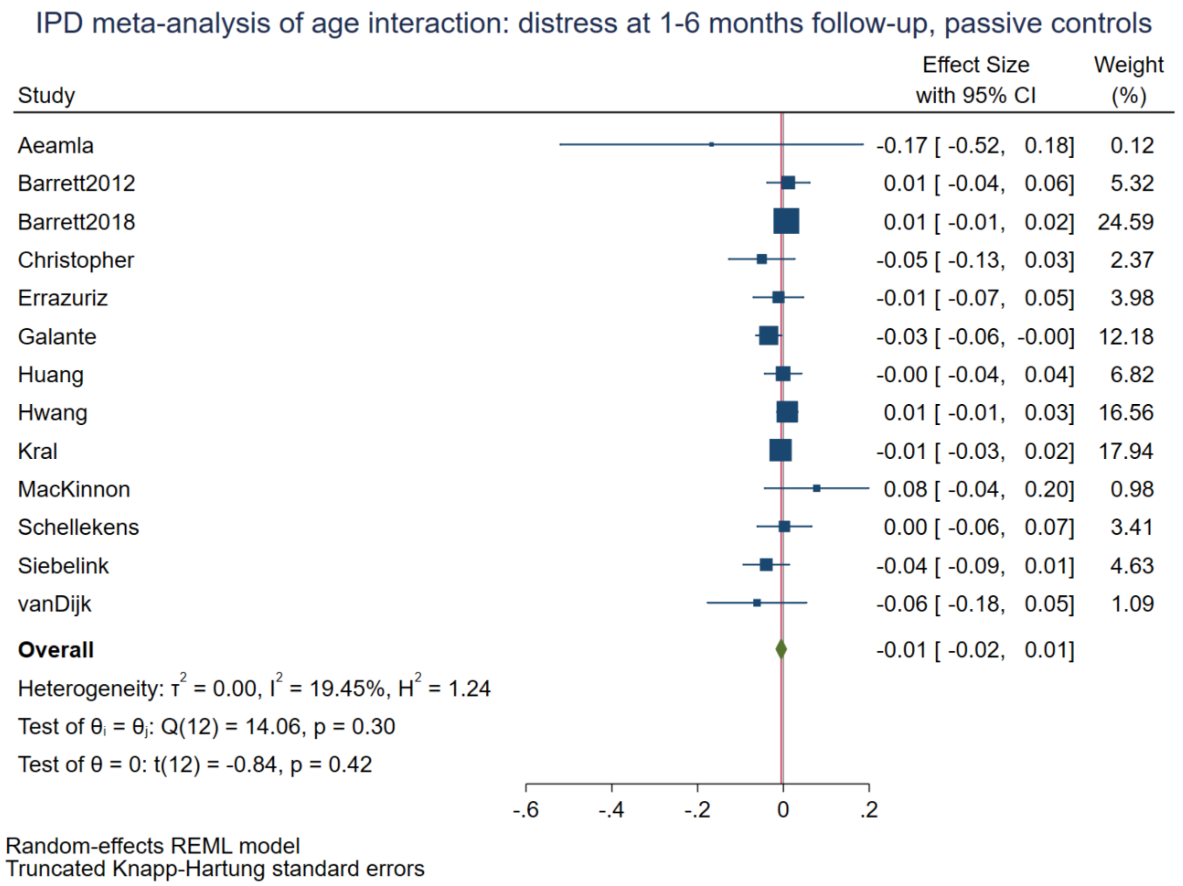

Supplementary Figure 4. Individual participant meta-analysis of age interaction: distress at 1-6 months follow-up, passive controls. Random-effects meta-analysis using the restricted maximum likelihood method (two-sided test with no adjustment for multiple comparisons). N= 2,371 participants. Data are presented as standardised mean differences (SMD) with 95% confidence intervals (CI).

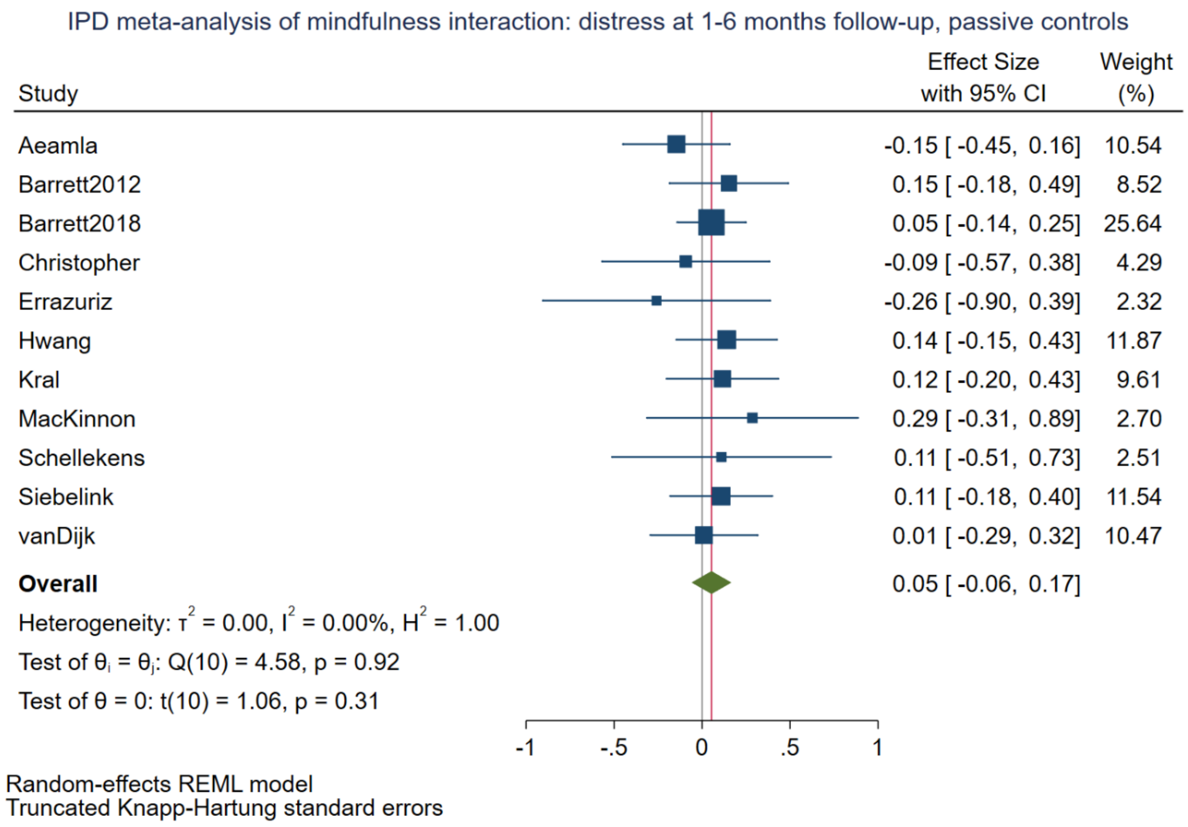

Supplementary Figure 5. Individual participant meta-analysis of mindfulness interaction: distress at 1-6 months follow-up, passive controls. Random-effects meta-analysis using the restricted maximum likelihood method (two-sided test with no adjustment for multiple comparisons). N= 1,557 participants. Data are presented as standardised mean differences (SMD) with 95% confidence intervals (CI).
